# Towards the Next Generation of Data‐Driven Therapeutics Using Spatially Resolved Single‐Cell Technologies and Generative AI

**DOI:** 10.1002/eji.202451234

**Published:** 2025-02-18

**Authors:** Avital Rodov, Hosna Baniadam, Robert Zeiser, Ido Amit, Nir Yosef, Tobias Wertheimer, Florian Ingelfinger

**Affiliations:** ^1^ Department of Systems Immunology Weizmann Institute of Science Rehovot Israel; ^2^ European Molecular Biology Laboratory Heidelberg Germany; ^3^ Department of Internal Medicine I Medical Center‐University of Freiburg Freiburg Germany

**Keywords:** autoimmunity, generative AI, single‐cell multi‐omics, spatial‐omics, variational autoencoders

## Abstract

Recent advances in multi‐omics and spatially resolved single‐cell technologies have revolutionised our ability to profile millions of cellular states, offering unprecedented opportunities to understand the complex molecular landscapes of human tissues in both health and disease. These developments hold immense potential for precision medicine, particularly in the rational design of novel therapeutics for treating inflammatory and autoimmune diseases. However, the vast, high‐dimensional data generated by these technologies present significant analytical challenges, such as distinguishing technical variation from biological variation or defining relevant questions that leverage the added spatial dimension to improve our understanding of tissue organisation. Generative artificial intelligence (AI), specifically variational autoencoder‐ or transformer‐based latent variable models, provides a powerful and flexible approach to addressing these challenges. These models make inferences about a cell's intrinsic state by effectively identifying complex patterns, reducing data dimensionality and modelling the biological variability in single‐cell datasets. This review explores the current landscape of single‐cell and spatial multi‐omics technologies, the application of generative AI in data analysis and modelling and their transformative impact on our understanding of autoimmune diseases. By combining spatial and single‐cell data with advanced AI methodologies, we highlight novel insights into the pathogenesis of autoimmune disorders and outline future directions for leveraging these technologies to achieve the goal of AI‐powered personalised medicine.

## Graphical abstract

This review explores the technical advances in single‐cell multi‐omics technologies alongside the parallel development of generative AI tools to uncover biological insights. By integrating these innovative approaches, we highlight the potential to identify diagnostic, prognostic and outcome biomarkers, enhance patient stratification systems and discuss future prospects for advancing precision medicine and personalised therapeutic strategies.

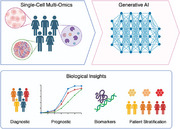

## 1 Introduction

Single‐cell multi‐omics have become routine assays for the exploratory analysis of heterogeneous samples in both preclinical and clinical research, enabling an unprecedented interrogation of virtually every cell type in the human body [1]. Large‐scale community efforts towards data reuse and standardisation, as well as advances in *data integration* [2, 3], have made it simpler and faster to query protein and gene expression in individual cells across species, organs and conditions, such as disease. This enormous and ever‐growing collection of molecular and cellular data holds great potential for improving diagnostics and therapeutic development, including the *rational design* of novel treatments, the prediction of side effects and a deeper understanding of disease‐driving processes [4]. The emergence of spatial assays to profile cells in situ, such as imaging‐ or sequencing‐based spatial transcriptomics, further bears the potential to generate transformative insights about niches, tissues and higher‐level organ organisation and uncover the underlying cellular communication networks [5]. While these technical developments yielded an unprecedented quantity and quality of cellular data, modern computational biology faces the challenge of developing methods that translate these complex high‐dimensional measurements into biological insights that benefit patients. *Generative AI*, and in particular *transformer*‐based deep *neural networks*, have already revolutionised the fields of natural language processing and computer vision by effectively identifying complex patterns in input data and capturing context‐awareness between related input elements [6]. These powerful *deep learning* architectures are currently being applied to single‐cell data with the aim of deciphering the ‘language’ of genes, cells and ultimately organisms and diseases.

Here, we review current developments and concomitant challenges of spatially resolved single‐cell technologies, discuss the potential of deep generative modelling to mitigate analytical bottlenecks and highlight transformative studies that have applied these techniques to advance our understanding of the pathogenesis of autoimmune disorders. Lastly, we will outline the next steps required to deliver the promised benefit of spatially resolved single‐cell technologies to the clinics.

## 2 Cellular Cartography: Navigating the Terrain of Single‐Cell and Spatial Omics

**FIGURE 1:**
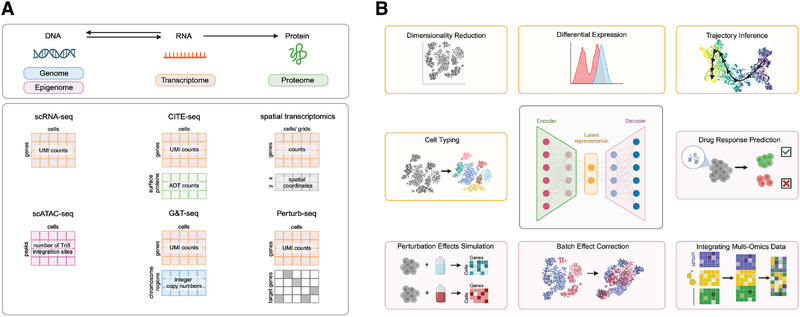
(A) Single‐cell multi‐omics provide a more comprehensive view of the cell by profiling different layers of the central dogma of biology. After initial preprocessing of single‐cell RNA‐seq (scRNA‐seq) fastq files, a matrix of UMI counts is generated for each gene in every cell. CITE‐seq enhances this by adding a proteomics layer, resulting in a matrix of antibody‐derived tags (ADT) counts per cell. Spatial transcriptomics provides gene expression data along with spatial coordinates of cells or grids, depending on the technology used. Single‐cell ATAC‐seq profiles the epigenome by reporting the number of Tn5 integration sites per peak. G&T‐seq integrates genomic and transcriptomic data by providing copy number variations across chromosome regions. Perturb‐seq adds a functional dimension by providing UMI counts in addition to the identity of perturbed genes in each cell. (B) The central VAE scheme illustrates how latent representation and decoded expression data can be utilised in single‐cell and spatial transcriptomics. Tasks framed in yellow are primarily derived from the latent space, focusing on data interpretation and dimensionality reduction, while tasks in pink are driven by the decoder, emphasising predictive and integrative capabilities.

Over the decades, technological advancements have significantly refined our ability to measure gene expression, progressing from early hybridisation techniques like *northern blotting* [7] to high‐throughput methods such as *DNA microarrays* [8], bulk RNA sequencing [9] and most recently, single cell RNA sequencing (scRNA‐seq). Unlike Northern blots and microarrays, which require prior knowledge of the genes to be assessed, bulk and scRNA‐seq aim to capture and profile the entire mRNA content in a sample, providing a more comprehensive and unbiased gene expression analysis. While bulk RNA‐seq offers an average gene expression profile across a large population of cells, scRNA‐seq further refines this by capturing gene expression at the individual cell level [10]. Since the conception of scRNA‐seq in 2009, various technologies have been developed, differing in single‐cell encapsulation protocols, capture of mRNA content in each cell, library preparation and sequencing [11]. These technologies, along with the practical considerations regarding throughput, sensitivity and cost, have been reviewed elsewhere [12, 13, 14, 15]. The raw output of scRNA‐seq consists of millions of sequencing reads, each containing one or more barcodes and a gene fragment. These reads are processed using standardised bioinformatics pipelines to generate matrices of *Unique Molecular Identifier (UMI)* counts for each gene in each cell (Figure 1A). Applying downstream analysis tools, cell types can be annotated and visualised in a *low‐dimensional embedding* [16, 17]. The standardised analysis workflow and best practices for each step have been extensively reviewed elsewhere [18, 19, 20, 21, 22].

ScRNA‐seq provides a wealth of new insights into cellular biology. One major advantage is the ability to discover new or rare cell types and cell states [23]. Furthermore, cell type composition can be compared across samples, tissues and conditions, shedding light on complex disease mechanisms and inter‐patient heterogeneity [24, 25]. Additionally, comparing gene expression profiles between conditions, complemented with *gene set enrichment analysis*, can identify pathways with differential activation levels [26]. Several tools have been developed to infer *gene regulatory networks* [27] or predict cell–cell communication events [28] from scRNA‐seq data, leveraging prior knowledge from databases on signalling pathways and cellular interactions. Another significant application is the alignment of cells along developmental or functional trajectories [29], the probabilistic modelling of cell fate [30] and the measurement of *developmental potential* [31]. This has provided insights into dynamic processes such as cell differentiation during embryonic development [32] and immune response [33], as well as the evolution of cancer cells during tumorigenesis and immune escape [34]. Finally, advances in the standardisation and acceleration of scRNA‐seq data generation and analysis have facilitated the creation of comprehensive cell atlases, mapping out the diverse cell types and states within tissues. These atlases serve as invaluable reference tools for identifying cell types and characterising transcriptional signatures [35, 36].

Profiling the transcriptome of individual cells has rapidly become a widely accessible method for assessing a cell's intrinsic state and its biological functions, significantly advancing our understanding of tissues and organisms in a largely unbiased manner. Recent technological innovations have expanded this approach by enabling the profiling of additional modalities. This involves considering the interactive molecular hierarchy of different omics layers—the genome, epigenome, transcriptome and proteome—as well as the influence of the cell's microenvironment through physical or chemical signalling (Figure 1A). A notable example is scATAC‐seq, which measures chromatin accessibility at single‐cell level [37, 38]. Single‐cell multi‐omics technologies take this a step further by measuring several modalities simultaneously within a cell, usually in addition to the transcriptome [5]. Prominent examples include CITE‐seq, which measures gene expression within each cell in addition to a selected set of surface protein markers [39], and G&T‐seq, which sequences genomic DNA and full‐length mRNA from single cells [40].

In the past, the spatial location of a cell within a tissue was largely overlooked due to the tissue dissociation process used in single‐cell assays. However, advances in spatial single‐cell transcriptomics now allow gene expression to be measured in situ [41, 42]. Guidelines on choosing the most suitable platform in terms of throughput, resolution, sensitivity and cost, in addition to standardised computational data analysis workflows, have been reviewed elsewhere [43, 44, 45, 46]. Studying the spatial coordinates of cells within a tissue, in addition to their gene expression profiles, enables the identification of *spatially variable genes*, spatial interactions between cells and multicellular niches with coregulated gene expression patterns [47].

Beyond traditional omics layers and the spatial location of cells, several innovative approaches integrate other biological and functional dimensions with transcriptomics to gain deeper insights into cellular functions and interactions. For example, Perturb‐seq combines *CRISPR‐based genetic screening* with single‐cell transcriptomics, allowing researchers to directly link genetic perturbations to their transcriptional effects, thus elucidating gene function and regulatory networks [48]. Additionally, lineage tracing techniques have been integrated with transcriptomics to track cell lineage and differentiation pathways over time, providing insights into developmental processes and tumour evolution [49]. V(D)J sequencing combined with transcriptomics offers a powerful tool for analysing T and B cell receptor repertoires, providing insights into clonality, immune diversity and responses at the single‐cell level [50]. Finally, by introducing fluorescent in vivo time stamps, Zman‐seq enables the recording of transcriptomic dynamics across time [51].

Single‐cell multi‐omics and spatial technologies have produced vast datasets, revealing new insights into cell types, states, interactions and disease mechanisms. The unbiased nature of these technologies has transformed experimental design, shifting the focus from hypothesis‐driven approaches to more exploratory data analysis. While this increases the potential for unexpected discoveries, it also presents challenges for reproducibility and statistical analysis. As costs decrease and the throughput of these technologies improves, along with the standardisation of computational tools, researchers will work with even larger datasets, offering a deeper understanding of disease. *Machine learning* and AI will play a crucial role in identifying *biomarkers* linked to disease and therapeutic responses, advancing precision medicine.

## 3 Generative Modelling Provides a Flexible Toolkit to Deliver Biologically Relevant Insights

The rise of single‐cell and spatial multi‐omics technologies has dramatically expanded our ability to explore novel biological questions that were previously unattainable [52]. However, these advancements come with significant analytical challenges, including the need to process vast datasets comprising hundreds of thousands of cells, to account for technical variations across different technologies and protocols and to manage *transcriptional stochasticity* [53]. To address these issues, the use of flexible and robust analytical tools is indispensable [54].

### 3.1 Latent Variable Models: Unveiling Cell States and Biological Variation With VAEs

Neural network–based *latent variable models* are computational models that utilise neural networks to uncover hidden structures and patterns within complex data. These models, including *Variational Autoencoders* (VAEs), excel at capturing intricate, non‐linear relationships in data, making them well‐suited for the integration and analysis of high‐dimensional single‐cell datasets (Figure 1B). VAEs are particularly valuable for reducing data complexity by generating biologically meaningful, low‐dimensional representations that reflect a cell's intrinsic state [55]. By decoupling biological variation from technical noise, they provide a well‐established statistical framework for evaluating uncertainty in measurements, which is especially relevant in single‐cell data where noise, sparsity and bias are prevalent. These models enable automated cell typing by querying reference atlases [55] or the identification of cellular states associated with disease or treatment responses [56, 57].

Latent variable models employing a VAE architecture, such as scVI (single‐cell variational inference), enable *probabilistic inference* of a cell's intrinsic state in single‐cell transcriptomics data (Table 1). This approach has proven particularly effective for modelling single‐cell genomics data and outperforms linear methods in atlas‐level integration tasks while maintaining biological variance [2]. Additionally, the generative model can be utilised for differential gene expression analysis, controlling for technical nuisance factors, thereby enhancing the accuracy of downstream analysis tasks such as clustering, transcription factor inference or pathway analysis. By incorporating additional latent space vector arithmetics, scGen [58] has demonstrated the ability to predict cellular responses to perturbations and isolate perturbation effects in a mixture of cell types. To address increased complexity in single‐cell transcriptomic studies, MrVI [59] (multiple‐resolution variational inference) leverages two distinct *latent spaces*: one representing within‐sample variation and the other capturing between‐sample variation, such as disease presence or treatment response (Table 1). This approach avoids the need to annotate predefined clusters, enabling the detection of subtle differences in cellular states and providing a nuanced understanding of gene expression and cellular composition across conditions.

**TABLE 1:** The table provides a comparative overview of model‐centric computational tools and platforms used in single‐cell and multi‐omic data analysis. It outlines each tool's architecture, such as variational autoencoders or transformers, and specifies the omic modalities they handle, like single‐cell or spatial transcriptomics. Additionally, the table summarises the main tasks these tools are designed to accomplish, offering a concise reference for their application in analysing complex biological data.

Tool/platform	Architecture	Modalities (**Figure** **1A**)	Main task (**Figure** **1B**)
scVI	Variational autoencoder	scRNA‐seq	Dimensionality reduction, batch effect correction, differential expression
scGen	Variational autoencoder	scRNA‐seq	Perturbation effects simulation
MrVI	Variational autoencoder	scRNA‐seq	Differential expression: gene expression analysis between conditions of interest. Cluster‐free differential abundance
VEGA	Variational autoencoder	scRNA‐seq	Drug response prediction: predict cell‐specific reactions to treatments. Latent variable interpretability.
LDVAE	Linear decoded variational autoencoder	scRNA‐seq	Dimensionality reduction: generation of interpretable gene modules
siVAE	Variational autoencoder	scRNA‐seq	Cell typing: identification of gene modules and hubs
DeepT2Vec	Autoencoder	Multi‐omic data	Cell typing: differentiation of cell types. Analysis of small sample sizes
COVET+ ENVI	Variational autoencoder	scRNA‐seq and spatial transcriptomics	Integrating multi‐omics data, analysing cellular interactions within specific tissue environments or niches
Tangram	Probabilistic generative framework	scRNA‐seq and spatial transcriptomics	Alignment of single‐cell data to spatial data, reconstruction of spatial gene expression
DeepST	Graph neural network autoencod	Spatial transcriptomics	Integration of spatial transcriptomics data, imputation of missing data.
Large‐scale foundation models: scGPT, geneformer, SiGra, scBERT	Transformer	Multi‐omic data	Integration and analysis of large complex datasets, pattern identification
Hugging face	Various deep learning architectures	Multi‐omic data	Develop, reuse and train machine learning models
CellxGene	Interactive visualisation platform	scRNA‐seq	Visualisation, finding, exploration, and analysis of single‐cell RNA‐seq datasets
scArches	Variational autoencoder + transfer learning	scRNA‐seq	Annotation of single‐cell datasets using a reference atlas

Deep neural network–based models have been developed to address specialised tasks in single‐cell analyses. For instance, VEGA [60] (VAE enhanced by gene annotations) uses user‐provided gene modules to enhance latent variable interpretability and uncover cell‐specific reactions to treatments and state identities. As highlighted in Table 1, LDVAE [61] addresses a similar task of improving the interpretation of latent dimensions by enabling the reconstruction of gene expression patterns and cellular states, making it easier to understand how different latent variables represent biological variation. DeepT2Vec [62] illustrates a different analytical strategy by merging unsupervised autoencoders with known supervised information, such as cell type [63]. This approach has proven effective in distinguishing cancers, normal tissues and various other traits, even when working with limited sample sizes. Similarly, scANVI [64] employs labels in a semi‐supervised manner to annotate datasets of unlabelled cells from manually annotated atlases.

### 3.2 Extending Generative AI to Decode Spatial Interactions

The application of generative AI models extends beyond single‐cell technologies involving dissociated cells. Spatial technologies have the potential to revolutionise our ability to uncover cellular interactions, niche compositions [65] and enhance our understanding of how cells are organised in situ [66]. However, these advancements introduce new analytical challenges. Imaging‐based spatial transcriptomics technologies, which profile a predefined panel of genes, are often limited by low gene coverage, while sequencing‐based methods struggle with overt data sparsity [67]. Additionally, technical variations arising from data generated from multiple slides, as well as errors in segmentation masks, pose specific challenges for the analysis and interpretation of spatial transcriptomics data. To tackle these novel data analytical challenges, COVET [68] (Covariance Environment) has been proposed. COVET is designed to capture gene interactions within tissue environments by analysing gene co‐variation across cells. When paired with the Environmental Variational Inference (ENVI) algorithm, COVET provides a robust framework for integrating spatial data, offering valuable insights into the spatial context of cellular neighbourhoods (Table 1). While COVET and ENVI are among the first tools to explicitly model gene co‐variation in spatial transcriptomics, they build upon recent advancements in generative AI applied to spatial technologies, such as Tangram [69] and DeepST [70], which focus on reconstructing spatial gene expression and imputing missing data (Table 1).

### 3.3 Transformer‐Based Foundation Models for Context Awareness

Nicheformer [71] is part of a novel class of large‐scale *foundation models*, which are computational models trained on large amounts of diverse data that have recently gained attention for their potential to improve our understanding of how cells interact and function within their surrounding environments [72]. These models are powered by transformer architectures, a type of neural network designed to handle sequential data by focusing on important parts of the input through a mechanism called ‘attention.’ Unlike simpler models like VAEs, transformers use the attention mechanism to dynamically weigh the importance of different inputs, making them particularly adept at modelling context awareness of input features. This ability is crucial in spatial transcriptomics, where expression of the same gene can have distinct biological consequences depending on the cellular context. For example, TGF‐β, a multifunctional cytokine, ensures microglial homeostasis in the central nervous system, suppressing pro‐inflammatory responses while promoting neuronal integrity [73]. In lymph nodes, TGF‐β preconditions naïve CD8+ T cells by epigenetically priming them for tissue‐resident memory differentiation, a process critical for immune surveillance in barrier tissues [74]. By capturing these long‐range dependencies, transformers not only provide new insights into the spatial organisation and functional relationships of cells within tissues but also offer significant opportunities for understanding the ‘language’ of genes. As summarised in Table 1, tools such as scGPT [75], Geneformer [76], SiGra [77] and scBERT [78], which are primarily designed for single‐cell RNA sequencing data analysis, demonstrate the power of transformers in querying cell types, imputing gene expression [79] and mapping tissue structures and cellular niches in greater detail.

### 3.4 Towards a Model‐Centric Future

Beyond the numerous ways generative AI models can address fundamental biological questions and accelerate data analysis, platforms like Hugging Face [80, 81] exemplify a community shift from a data‐centric to a model‐centric approach. This paradigm emphasises the development, sharing and reuse of models for biological data analysis, moving beyond merely accumulating large datasets. A key aspect of this model‐centric approach is *transfer learning*, which enables models trained on large datasets to be fine‐tuned for specific tasks with smaller datasets, enhancing their versatility and efficiency. This allows researchers to leverage pretrained models to quickly adapt to new research questions or experimental conditions without the need to store large amounts of data, significantly speeding up discovery and reducing the resource burden. For instance, scArches [82] uses transfer learning to adapt models for single‐cell data across different conditions and technologies.

Looking ahead, developing an ecosystem that adheres to FAIR (findable, accessible, interoperable and reusable) principles will be essential for sustaining the model‐centric approach. Models should be shared through user‐friendly repositories like Hugging Face, which provide clear details about model tasks, training data and usage requirements, ensuring accessibility. Sharing lightweight pretrained models, rather than large raw datasets like count matrices, reduces resource demands and enables equitable access, empowering researchers in diverse settings to benefit from cutting‐edge tools.

Thanks to the community‐driven efforts, along with the availability of training resources, comprehensive documentation and user‐friendly vignettes, these advanced deep learning tools—despite their mathematical complexity—are becoming more accessible to researchers across various fields, including immunology. This increased accessibility allows us to fully leverage these cutting‐edge technologies, with the potential to revolutionise our understanding of biology, facilitating breakthroughs in personalised medicine, predictive modelling of disease progression and the design of novel therapeutics.

## 4 Transformative Insights Into Autoimmune Disease Mechanisms Through Single‐Cell and Spatial Transcriptomics

The advent of the aforementioned single‐cell and spatial transcriptomics technologies, combined with advanced computational frameworks, holds unprecedented potential to untangle the cellular interactions and molecular programs underlying immune‐mediated diseases. In recent years, numerous studies have mapped the single‐cell landscapes of human autoimmune or immune‐mediated diseases, including rheumatoid arthritis (RA), Type 1 Diabetes, multiple sclerosis (MS) and inflammatory bowel diseases (IBD) [83, 84, 85, 86]. RA, a highly prevalent autoimmune disorder affecting approximately 1% of the global population, is primarily characterised by synovial inflammation, which progressively leads to cartilage destruction and a broad spectrum of extra‐articular manifestations [87]. Recent single‐cell studies have significantly expanded our understanding of immune cell heterogeneity in RA, revealing distinct pathogenic states in T cells, B cells, monocytes and fibroblasts. For example, peripheral helper T cells producing CXCL13 and IL‐21 are linked to B‐cell activation and autoantibody production, with their abundance correlating with treatment resistance [88]. Similarly, THY1^+^HLA‐DR^hi^ sublining fibroblasts are major producers of IL‐6 and CXCL12 and are enriched in leukocyte‐rich RA synovial tissue, contributing to local inflammation. Additionally, IL1B^+^ monocytes have been identified as key pro‐inflammatory mediators, showing elevated expression of genes such as NR4A2 and PLAUR, which are associated with cytokine activation and matrix remodelling in active RA joints [25]. Subsequent work has laid the groundwork for developing tissue classification systems based on distinct cellular states in RA patients, laying the foundation for targeted therapies tailored to specific cellular compositions within the RA synovium [88]. In this seminal study, a comprehensive multi‐modal atlas of RA synovial tissue from 79 patients was created using scRNA‐seq, surface protein profiling and histology. The study identified six distinct Cell‐Type Abundance Phenotypes (CTAPs), each enriched in specific cell lineages such as T and B cells, fibroblasts or myeloid cells. These CTAPs underscore the heterogeneity of synovial inflammation in RA. Notably, certain CTAPs, such as those enriched in T peripheral helper cells and inflammatory fibroblast subsets like NOTCH3^+^ fibroblasts, correlate with histological features and treatment responses, demonstrating their clinical utility in stratifying patients and predicting therapeutic outcomes. The mechanistic insights gained from these studies are not limited to classical autoimmune diseases but also extend to conditions like immune‐related adverse events (ir‐AEs), which affect patients undergoing cancer immunotherapies [89].

Since spatial context is crucial for deciphering the cellular communication circuits driving immunopathology, spatial transcriptomics and proteomics platforms have gained increasing attention for their ability to deepen our understanding of cellular interactions in inflammatory diseases. For example, in preclinical models of IBD, spatial transcriptomics using MERFISH has suggested a staged progression of inflammation‐associated tissue neighbourhoods, characterised by inflammation‐associated fibroblasts with distinct spatial localisations and communication axes [90]. Similarly, in MS, spatial transcriptomics has provided valuable insights into the evolving cellular architecture of neuroinflammatory lesions. By performing in situ sequencing in both experimental autoimmune encephalomyelitis and human MS samples, a recent study revealed dynamic changes in glial states and their spatial preferences, which are critical for understanding lesion compartmentalisation and disease progression [91]. This work demonstrated the centrifugal evolution of active lesions, showing that disease‐associated (DA) glial cells are dynamically induced and resolved independently of lesion formation. For instance, DA oligodendrocytes were observed to express MHC class I and II molecules, enabling antigen presentation and T cell activation. Additionally, spatial mapping highlighted distinct sub‐compartments within lesions, such as lesion rims enriched with DA microglia and astrocytes, which interact to maintain the inflammatory microenvironment. Human MS spinal cord samples corroborated these findings, revealing compartment‐specific distributions of glial cells and immune infiltrates that correlate with lesion activity and chronicity. These insights underscore the role of spatial preferences in lesion evolution, offering potential targets for therapeutic intervention.

Building on these novel pathophysiological insights into cellular interactions and molecular programs, current research is leveraging multi‐omics single‐cell data and advanced computational tools to identify therapeutic targets and predict patient responses in inflammatory diseases. For instance, multi‐omics single‐cell data, combined with machine learning tools, have been used to predict patient responses to anti‐TNF treatments in RA [92]. This study proposed biomarkers, such as the CHI3L1 gene and its protein YKL‐40, which were suppressed in responders. Machine learning models based on baseline transcriptomic data achieved high predictive accuracy, highlighting the potential of multi‐omics approaches to guide personalised medicine in RA. Similarly, studies have investigated the underlying cellular and molecular correlates in patients with ir‐AEs using single‐cell technologies, proposing potential biomarkers to identify patients at risk or those who might benefit from early therapeutic intervention. In this regard, Núñez et al. identified an early expansion of Ki‐67^+^ regulatory T cells and proliferating CD8^+^ CD38^+^ Ki‐67^+^ T cells as predictive markers of ir‐AEs in patients treated with ICIs. These CD8^+^ T cells expressed activation markers, including Eomes and IFN‐γ, linking them to heightened immune activation. Increases in IFN‐γ‐driven cytokines, such as CXCL9, CXCL10 and CXCL11, were also associated with a higher risk of ir‐AEs. Lozano et al. further identified activated CD4^+^ effector memory T cells, characterised by markers such as HLA‐DRA and TNFRSF4 (OX40), as correlates of ir‐AE development. While immune activation pathways overlap between ir‐AEs and anti‐tumour responses, Núñez et al. demonstrated that early immune signatures, including IFN‐γ‐driven cytokines and Ki‐67^+^ T cell subsets, were more strongly associated with the development of ir‐AEs than with response to therapy. These findings underscore the complex interplay of immune mechanisms underlying toxicity and therapeutic efficacy and provide critical insights for risk stratification and personalised management. Another notable approach is the scDrugPrio framework, which uses scRNA‐seq data to systematically prioritise and repurpose drugs by mapping disease‐specific cellular states and molecular interactions [93]. Applied to conditions such as RA, MS and IBD, these computational tools are laying the foundation for more personalised treatment approaches. Despite these advancements, the large number of therapeutic agents available and the heterogeneous responses among patients highlight the need for even more sophisticated models. The emergence of transformer‐based foundation models offers a promising solution by identifying common cell states across various autoimmune conditions, predicting differential treatment responses and addressing the complex biological mechanisms driving disease manifestations. As these tools continue to evolve, we anticipate their growing relevance in clinical settings, translating our understanding of intricate pathogenic immunodynamics into effective, individualised treatment strategies.

## 5 Conclusions

Since the sequencing of the very first single cell in 2009, the field of biomedicine, particularly immunology, has experienced a technological revolution [10]. While the conceptual advancements in profiling millions of single cells in tissues through multi‐omics are undeniable, the practical utility of these technologies in improving patient care is yet to be fully demonstrated to justify the high financial and personal expenses associated with these technologies. This gap may be addressed by defining and contextualising novel biological questions, along with hypotheses that can be empirically tested using spatially resolved and multi‐omics single‐cell assays. Yet, the considerable costs of spatial transcriptomics and the need for specialised laboratory equipment largely impede the large‐scale, systematic collection of spatial transcriptomics data at clinical sites. Current community‐driven efforts, such as Open‐ST [94], have demonstrated the potential to reduce the cost of spatially resolved transcriptomics assays by an order of magnitude, thereby highlighting the role of non‐commercial initiatives in increasing the accessibility of these technologies beyond specialised or well‐funded laboratories. Large consortia projects, such as the tumour profiling study, have convincingly shown the potential of spatially resolved molecular profiling technologies in guiding clinical decision‐making, provided that requirements such as standardisation of protocols for data generation and analysis, short turnaround times and the construction of necessary infrastructure are met [95]. A recent study demonstrated the transformative impact of spatially resolved single‐cell technologies by identifying type 1 and 2 interferon signalling as a key molecular hallmark of toxic epidermal necrolysis and repurposed JAK inhibitors as a curative therapy for this fatal disease, for which no effective therapeutic options were previously available [96]. While seminal studies like these motivate the scientific community to increase the availability of spatially resolved single‐cell technologies in the clinics, it remains a matter of public debate to what extent the financial costs to healthcare services and the associated economic and environmental burdens justify the use of these technologies in routine clinical practice. The promise of generative AI is to replace many of the experimental procedures with in silico predictions to accurately infer the impact of any given drug on individual cellular states, intact tissues and even entire organisms. Therefore, the next step in systems biomedicine is to collect sufficient data and build adequately powered models to achieve the ultimate goal of selecting the best possible therapy regimen for any given patient and enter an era of AI‐powered personalised medicine.

## Conflicts of Interest

The authors declare no conflicts of interest.

### Peer Review

The peer review history for this article is available at https://publons.com/publon/10.1002/eji.202451234

## Data Availability

The authors have nothing to report.
